# Silver Nanoparticles Formulation of Flower Head’s Polyphenols of *Cynara scolymus* L.: A Promising Candidate against Prostate (PC-3) Cancer Cell Line through Apoptosis Activation

**DOI:** 10.3390/molecules27196304

**Published:** 2022-09-24

**Authors:** Amgad I. M. Khedr, Marwa S. Goda, Abdelaziz F. S. Farrag, Ali M. Nasr, Shady A. Swidan, Mohamed S. Nafie, Maged S. Abdel-Kader, Jihan M. Badr, Reda F. A. Abdelhameed

**Affiliations:** 1 Department of Pharmacognosy, Faculty of Pharmacy, Port Said University, Port Said 42526, Egypt; 2 Department of Pharmacognosy, Faculty of Pharmacy, Suez Canal University, Ismailia 41522, Egypt; 3 Department of Pharmacognosy, Faculty of Pharmacy, Galala University, New Galala 43713, Egypt; 4 Department of Pharmaceutics, Faculty of Pharmacy, Port Said University, Port Said 42526, Egypt; 5 Department of Pharmaceutics and Pharmaceutical Technology, Faculty of Pharmacy, The British University in Egypt, El-Sherouk City 11837, Egypt; 6 The Centre for Drug Research and Development (CDRD), Faculty of Pharmacy, The British University in Egypt, El-Sherouk City 11837, Egypt; 7 Department of Chemistry, Faculty of Science, Suez Canal University, Ismailia 41522, Egypt; 8 Department of Pharmacognosy, College of Pharmacy, Prince Sattam Bin Abdulaziz University, Al-Kharj 11942, Saudi Arabia; 9 Department of Pharmacognosy, Faculty of Pharmacy, Alexandria University, Alexandria 21215, Egypt

**Keywords:** *Cynara scolymus* L., antioxidant activity, total phenolic, HPLC-DAD, flower, bract, stem, silver nanoparticles, PC-3, A549

## Abstract

*Cynara scolymus* L. (Family: Compositae) or artichoke is a nutritious edible plant widely used for its hepatoprotective effect. Crude extracts of flower, bract, and stem were prepared and evaluated for their in vitro antioxidant activity and phenolic content. The flower crude extract exhibited the highest phenolic content (74.29 mg GAE/gm) as well as the best in vitro antioxidant activity using total antioxidant capacity (TAC), ferric reducing antioxidant power (FEAP), and 1,1-diphenyl-2-picrylhyazyl (DPPH) scavenging assays compared with ascorbic acid. Phenolic fractions of the crude extracts of different parts were separated and identified using high-performance liquid chromatography HPLC-DAD analysis. The silver nanoparticles of these phenolic fractions were established and tested for their cytotoxicity and apoptotic activity. Results showed that silver nanoparticles of a polyphenolic fraction of flower extract (Nano-TP/Flowers) exhibited potent cytotoxicity against prostate (PC-3) and lung (A549) cancer cell lines with IC_50_ values of 0.85 μg/mL and 0.94 μg/mL, respectively, compared with doxorubicin as a standard. For apoptosis-induction, Nano-TP/Flowers exhibited apoptosis in PC-3 with a higher ratio than in A549 cells. It induced total prostate apoptotic cell death by 227-fold change while it induced apoptosis in A549 cells by 15.6-fold change. Nano-TP/Flowers upregulated both pro-apoptotic markers and downregulated the antiapoptotic genes using RT-PCR. Hence, this extract may serve as a promising source for anti-prostate cancer candidates.

## 1. Introduction

Globally, medicinal plants are widely used for the prevention or treatment of many illnesses with minimal or no side effects. Curing with herbal medicine is back to prehistoric times [1], and it is expressed in about 90% of traditional therapies [2]. So, many scientific papers were oriented to illustrate how these plants undergo their pharmacological effects. Previous studies involved green medicine as a crude extract of the whole plant [3,4], an extract of certain parts [5,6], purified natural compounds [7,8,9], or isolated certain bioactive fractions such as polyphenols, essential oils, saponins, alkaloids, polysaccharides, etc. [10,11,12,13,14].

Plant-based foods naturally comprise polyphenolic compounds. Polyphenols are well-known for their free radical scavenging, antioxidant, and anti-carcinogenic activities. Artichoke or *Cynara scolymus* L. (Family: Compositae) is an edible plant famous for its hepatoprotective, antioxidant, antiviral, hypoglycemic, anti-hypercholesterolemic, antimicrobial, chemopreventive, and cytotoxic effects [15,16,17]. *C. scolymus* L. is traditionally consumed in the Mediterranean diet. The edible parts are a flower head (inflorescence) surrounded by fleshy leaves called bracts, and the proximal part of the stem [18]. Despite its low caloric value, it is a nutritious source of carbohydrates (6.8%), nitrogen compounds (2.9%), inulin (19–36%), fiber, vitamin C, folates, vitamin B complex, potassium, calcium, sodium, magnesium, phosphorus, iron, copper, and manganese [18]. *C. scolymus* L. is an enriched source of polyphenolic compounds classified as flavonoids, phenolic acids, anthocyanins, and glycosides. These polyphenols were identified using liquid chromatography equipped with mass spectrometry as chlorogenic acid, ferulic acid, coumaric acid, syringic acid, dicaffeoylquinic acid, gallic acid, rosmarinic acid, cyanidin 3,5-diglucoside, cyanidin 3-glucoside, luteolin, rutin, quercetin, naringenin, chrysoeriol, isoquercitrin, cynarin, and cynaroside [19,20]. Although artichoke was reported as traditional phytotherapy for the treatment of many diseases, few clinical trials about *C. scolymus* L. were provided. A previous study demonstrated the positive effect of *C. scolymus* L. on obesity treatment and lowering body mass index. *C. scolymus* L. can also control blood pressure in hypertensive patients through vasodilation and upregulation of endothelial-type nitric-oxide synthase gene expression [21]. Another study reported the ability of *C. scolymus* L. to reduce fasting blood glucose level, A1C-derived average glucose (ADAG), and homeostatic model assessment (HOMA), which assess β-cell function and insulin resistance, in prediabetic patients [22]. Fallah Huseini and his coworkers proved the antihyperlipidemic effect of *C. scolymus* L. via a reduction in total cholesterol level and low-density lipoprotein (LDL) [23]. All these findings pertained to existing polyphenolic compounds. Polyphenolic compounds exhibited a crucial impact in the treatment of cancer [24]. So, previous studies reported the cytotoxic effect of *C. scolymus* L. against different types of cancer such as cervical (HeLa) [25], breast (MCF-7) [26], liver (HepG-2) [27], and colon (HCT-116) cancer cell lines [28]. Prostate cancer is the fifth key reason of death around the world and the second most common tumor (after lung tumor) in men as well [29]. Therefore, the current study handled the efficacy of *C. scolymus* L. in the treatment of prostate cancer and lung cancer.

Nowadays, delivery systems of green medicine have been designed and developed as a result of recent breakthroughs in drug manufacturing. A nanocarrier of silver nanoparticles (AgNPs) is a sophisticated and well-designed system created for optimal delivery of biomaterials due to their surface-adsorbed molecules [30,31]. 

In view of the above, formulation of AgNPs of a phenolic portion of edible parts of flower, bract, and stem of *Cynara scolymus* L. (artichoke) is handled as a part of ongoing efforts to compare their cytotoxic activity versus their non-formulated crude extracts against lung and prostate cancers.

## 2. Results

### 2.1. In Vitro Antioxidant Activity of Crude Extracts of Flower, Bract, and Stem of C. scolymus *L.*

Artichoke is well-known for its antioxidant and free radical, and scavenging activities. The comparative in vitro antioxidant activity of the flower, leaf, and stem parts was assessed using three different methods, total antioxidant capacity (TAC) assay, ferric reducing antioxidant power (FEAP) assay, and 1,1-diphenyl-2-picrylhyazyl (DPPH) scavenging assay. From the data shown in Table 1, the crude extract of the flower part displayed the highest TAC. The TAC is defined as the total quantity of antioxidant which can react with an oxidant and is expressed as gallic acid equivalent per gram extract [32]. In addition, the flower extract exhibited a promising DPPH free radical scavenging activity and a potent ferric reducing power with IC_50_ of 45.91 and 64.39 µg/mL, respectively, compared with ascorbic acid as a reference drug. FRAP evaluates the potential of crude extracts of different parts of the plant to reduce ferric ion complex through the donation of an electron, while the DPPH assay evaluates the potential of crude extracts to donate an electron to neutralize DPPH free radicals [32]. The lower concentration of IC_50_ of the crude extracts under investigation, the better antioxidant potential. 

Stem extract showed the lowest value in the TAC assay. Additionally, neither FRAP nor DPPH scavenging activities of stem extract were observed.

### 2.2. Determination of Total Phenolics Content and Total Flavonoids Content in Crude Extracts of Flower, Bract, and Stem of C. scolymus *L.*

The antioxidant activity is highly correlated to phenolic compounds. In accordance with the results of the in vitro antioxidant assays, the crude extract of the flower part manifested the highest content of both flavonoids and phenolic compounds (Table 2). 

Of the three different extracts, it was noticed that flavonoid content in the flower is two-fold higher than that in bract and five-fold that in the stem, which contributes to the highest antioxidant activity of the flower. Moreover, the total phenolic content in the stem is half the amount of that found in bract and one third amount of that found in the flower, resulting in the lowest antioxidant activity as mentioned above. 

### 2.3. HPLC-DAD Identification of Polyphenols in Crude Extracts of Flower, Bract, and Stem of C. scolymus *L.*

**Figure 1:**
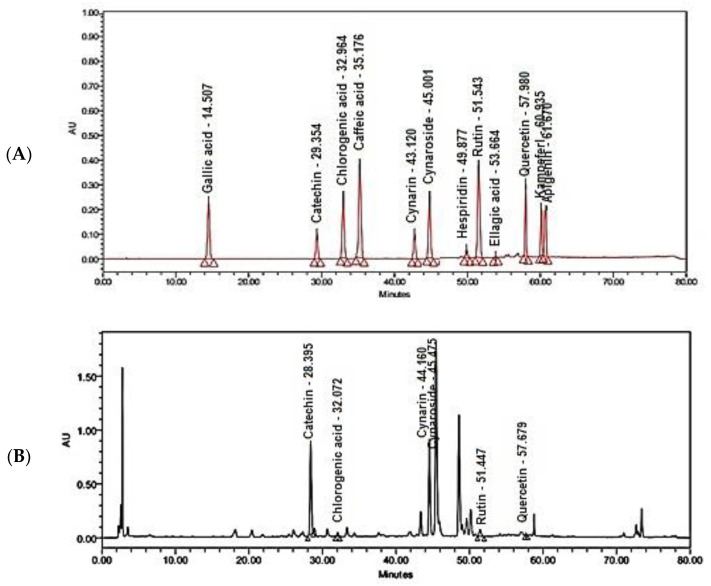

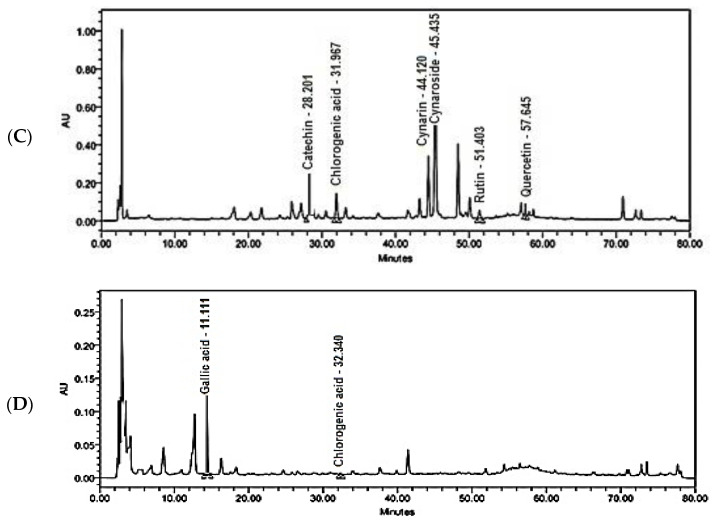
HPLC-DAD chromatograms: (**A**) Chromatogram of ten reference standards of polyphenols; (**B**) Chromatogram of polyphenols in flower crude extract; (**C**) Chromatogram of polyphenols in bract crude extract; (**D**) Chromatogram of polyphenols in stem crude extract.

A comparison of polyphenolic compounds in flower, stem, and bract extracts was conducted using HPLC-DAD analysis. On the one hand, the HPLC chromatogram of flower extract is closely related to that of the bract, showing almost the same major peaks (Figure 1). Among the investigated reference standards, the peaks of catechin, chlorogenic acid, cynarin, cymaroside, rutin, and quercetin were detected in both flower and bract extracts. Cynarin is the most commonly identified polyphenolic compound in flower and leaf extracts. On the other hand, the HPLC chromatogram of stem extract is poor with polyphenolic peaks. Only the peaks of gallic acid and chlorogenic acid were detected. Additionally, the peaks of ellagic acid, caffeic acid, hesperidin, and kaempferol are missing in all the represented chromatograms.

### 2.4. Characterization of AgNPs of Phenolic Portions of Crude Extracts of Flower, Bract, and Stem of C. scolymus *L.*

#### 2.4.1. UV-VIS Spectroscopy

**Figure 2:**
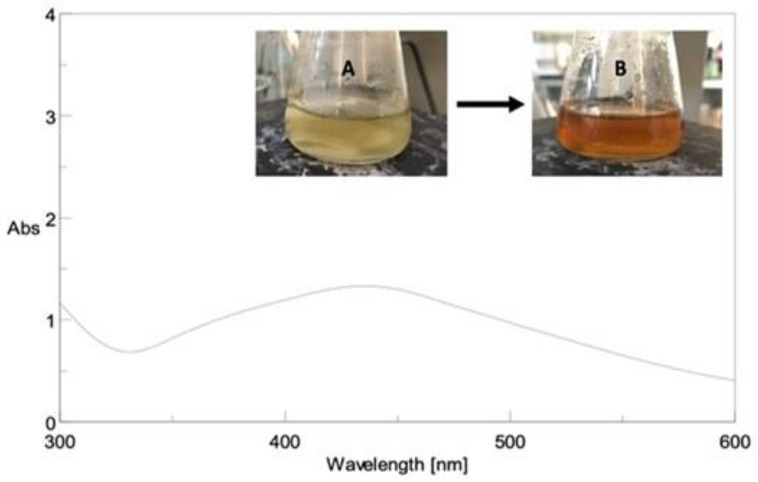
(**A**) The UV-visible spectrum of prepared AgNPs from the phenolic extract of flower part ethanolic extract; (**B**) AgNPs.

Due to the unique surface-plasmon resonance absorption band in the region of 400–500 nm, all AgNPs formulations developed a brown color, as shown in Figure 2.

#### 2.4.2. Transmission Electron Microscopy (TEM)

**Figure 3:**
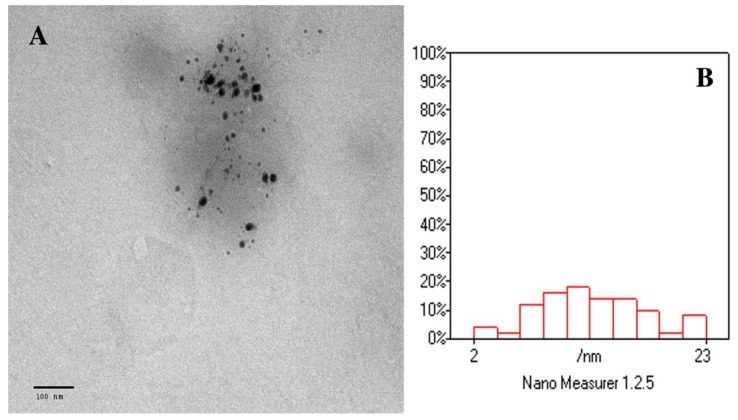
(**A**) TEM micrograph of total phenolics AgNPs of the flower. Mag. 120,000×; (**B**) TEM analysis using Nano Measurer software.

The total phenolics AgNPs of the flower are spherical in shape, fairly monodispersed, and well distributed without aggregation, according to the TEM image (Figure 3A). The particle size distribution of AgNPs generated from TEM data using Nano Measurer software is shown in Figure 3B.

#### 2.4.3. Particle Size and Zeta Potential Determination

The mean particle size values of the prepared AgNPs obtained ranged between 21.31 ± 0.43 and 26.42 ± 1.08 nm. The smallest particle size obtained using the DLS technique was of total phenolics AgNPs of the flower part (Nano-TP/Flower), but it is larger than that derived from the TEM image. This is expected as the DLS measures the hydrodynamic size, not the physical size [33]. Diegoli and coworkers mention that this difference is because the DLS technique is sensitive to the double layer surrounding the nanoparticles in dispersion, which is expected to lead to overestimating the mean particle diameter [34]. 

All generated AgNPs formulas had PDI values ranging from 0.101 ± 0.017 to 0.112 ± 0.020, exhibiting uniform size distribution and excellent homogeneity (Table 3). In addition, the zeta potential values of the prepared formulations ranged from −31.9 ± 2.22 to −35.5 ± 2.69 mV, as shown in Table 3. According to the ZP data, all generated AgNPs contain sufficient charges to prevent agglomeration and are regarded as highly stable.

### 2.5. In Vitro Cytotoxic Activity 

#### 2.5.1. In Vitro Cytotoxic Activity of Phenolic Fractions and Their AgNPs of Flower, Bract, and Stem against PC-3 and A549 Cell Lines

**Figure 4:**
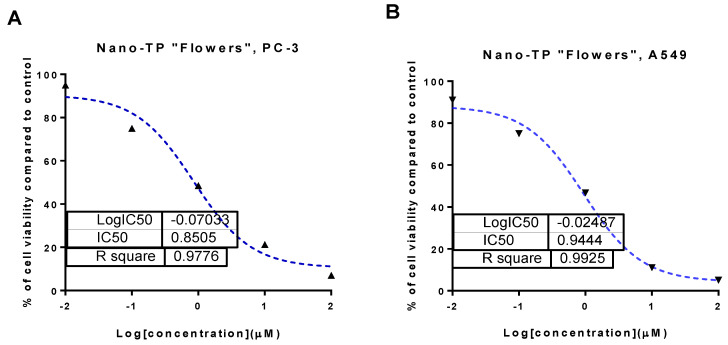
Percentage of cell viability vs. log [con. µM], R square ≈1 using the GraphPad prism software: (**A**) cytotoxicity of Nano-TP/Flowers against PC-3 cells; (**B**) cytotoxicity of Nano-TP/Flowers against A549 cells.

Samples of total phenolic fractions of different parts of *C. scolymus* L.; bract, flower, stem, and their AgNPs forms were screened for their cytotoxicity against prostate (PC-3) and lung (A549) cancer cell lines using MTT assay. As seen in Table 4, total phenolics of bracts, flowers, and stems exhibited moderate cytotoxic activity against PC-3 and A549 cell lines with an IC_50_ range of 16.35 to 56.3 μg/mL. Interestingly, AgNPs formulations improved the cytotoxicity results compared with their non-formulated forms. Nano-TP/Flowers displayed potent cytotoxicity against PC-3 and A549 cell lines with IC_50_ values of 0.85 μg/mL and 0.94 μg/mL, respectively (Figure 4), compared with doxorubicin with IC_50_ values of 5.13 and 6.19 μg/mL, respectively.

Additionally, Nano-TP/Bract showed potent cytotoxicity with IC_50_ values of 1.01 and 1.34 μg/mL. At the same time, Nano-TP/Stem exhibited moderate cytotoxicity with IC_50_ values of 14.3 and 13.6 μg/mL. These results highlighted the potent cytotoxicity of the Nano-TP/Flowers; hence it was worthy of being further tested for the mechanism of action in PC-3 and A549 cells.

#### 2.5.2. Apoptosis-Induction Activity

- Annexin V/PI staining

**Figure 5:**
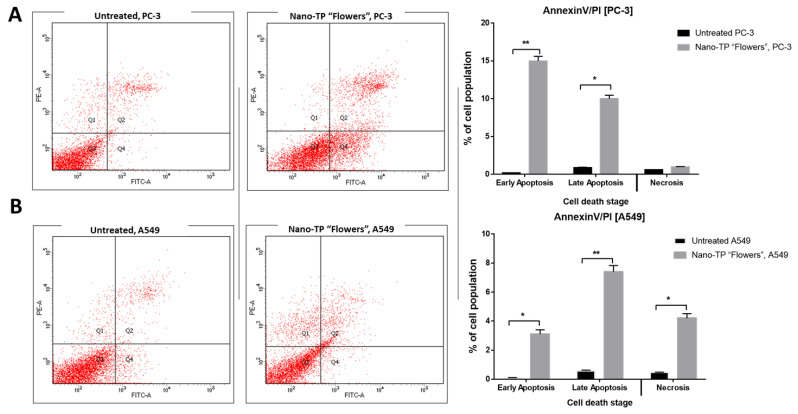
Cytograms and bar representation for apoptosis-necrosis assessment using flow cytometry: (**A**) Annexin V/PI staining of untreated and treated PC-3 cancer cells with Nano-TP/Flowers (IC_50_ = 0.94 µM, 48 h); (**B**) Annexin V/PI staining of untreated and treated A549 cancer cells with Nano-TP/Flowers (IC_50_ = 0.85 µM, 48 h). Q1: Necrosis, Q2: Late apoptosis, Q4: Early apoptosis. Lower panel. * (*p* ≤ 0.05) and ** (*p* ≤ 0.001) are significantly different using the unpaired test in GraphPad Prism.

Further investigation of the mechanism of apoptosis-induction of Nano-TP/Flowers against PC-3 and A549 cells was handled using Annexin V/PI staining. As seen in Figure 5, Nano-TP/Flowers induced total prostate apoptotic cell death by 25% (15% early and 10% late apoptosis) compared with 0.11% in the untreated control cells. So, it induced apoptosis in PC-3 cells by 227-fold change. Additionally, it induced total lung apoptotic cell death by 10.5% compared with 0.67% in the untreated control cells. So, it induced apoptosis in A549 cells by a 15.6-fold change. Consequently, Nano-TP/Flowers exhibited apoptosis in PC-3 with a higher ratio than in A549 cells.

- Gene expression analysis using RT-PCR

**Figure 6:**
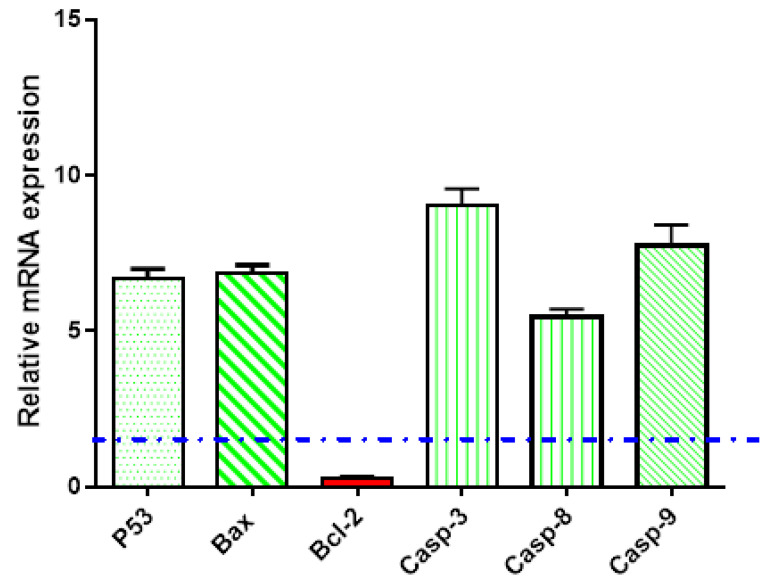
Gene expression analysis of untreated and treated PC-3 cells with Nano-TP/Flowers (IC_50_ = 0.85 µM, 48 h). β-actin was used as a housekeeping gene. Fold of change is calculated by 2^−ΔΔCT^, where ^ΔΔCT^ is the difference between mean values of genes CT values in the treated and control groups. Blue dashed line represents fold of change of untreated control = 1.

Gene expression analysis for the apoptosis-related genes was carried out in the untreated and treated PC-3 cells with Nano-TP/Flowers. As seen in Figure 6, Nano-TP/Flowers upregulated the P53 gene by 6.66-fold, the Bax gene by 6.88-fold, and caspases 3, 8, 9 by 9.05, 5.43, and 7.08-fold, respectively. While it downregulated the Bcl-2 gene by 0.25-fold, this behavior of apoptosis-induction in PC-3 cells upon treatment agreed with routine results of proving apoptosis-induction [35,36]. 

## 3. Discussion

*C. scolymus* L. is famous for its hepatoprotective effect as a result of having a high number of polyphenolic compounds. These polyphenols exhibited antioxidant and free radical scavenging activities that help in the prevention and treatment of many diseases. The total antioxidant capacity assay determines what number of free radicals or metal ions scavenged by the different samples [37]. On the one hand, DPPH has been widely used in the determination of the ability of polyphenolic compounds to capture free radicals. Free radicals are the root cause of cell damage, cancer, inflammation, aging, and other diseases. An advantage of the DPPH assay is that the free radical is commercially available and stable, and it doesnot generate in different ways [38]. On the other hand, the FRAP assay was developed as an alternative way to determine iron reduction in biological fluids [38]. Iron has an essential role in biochemical processes in our body. It is responsible for oxygen transport and storage, appropriate immune response, energy metabolism, and synthesis of collagen. The reduction of ferric iron is a key biological process during cellular iron uptake. So, it is a good way to assay the ability of polyphenolic compounds to reduce ferric iron that in high levels is involved in rusting blood vessels [39]. *C. scolymus* L. showed a positive effect on both mechanisms. Interestingly, flavonoids and phenolic acids are large groups of secondary metabolites that act as a donor of a hydrogen atom or an electron. The hydroxyl group at carbon number 3 in the flavonoid skeleton potentiates FRAP activity without any effect on DPPH scavenging activity. So, lacking this hydroxyl group in the structure of phenolic acids lessens FRAP activity [37]. The highest content of both total phenolics and total flavonoids was recorded in the crude extract of the flower part of *C. scolymus* L. As reported in the literature [19], *C. scolymus* L. is a fruitful source of phenolic compounds apart from flavonoids. Now, it is clear why the crude extract of the flower part of *C. scolymus* L. demonstrated a higher FRAP, DPPH scavenging activity, and TAC than these of bract or stem.

UPLC/MS-MS Analysis of some members of the family Asteraceae revealed the presence of high levels of luteolin-7-*O* glycoside (cynaroside) and apigenin-7-*O* glycoside as the most common flavonoids [40]. Concerning phenolic acids, chlorogenic acid is the most detected phenolic acid. An earlier study reported that caffeoyl derivatives are considered the major metabolite in the family Asteraceae, such as cynarin (dicaffeoylquinic acid) in artichoke and chicoric acid (dicaffeoyltartaric acid) in chicory [41]. The HPLC-DAD detection of polyphenols in flower, bract, and stem extracts was another comparative point in the current study. Our findings accord with those found in previous studies reporting the presence of chlorogenic acid, gallic acid, cynarin, cynaroside, rutin, and quercetin, as well as the absence of ellagic acid and hesperidin [19]. Unlike stem extract, the polyphenolic compounds in flower and bract extracts are almost the same. Different caffeoylquinic acid derivatives and flavonoids were previously isolated from leaf and flower parts, and then they were identified as chlorogenic acid, 1,3-di-*O*-caffeoylquinic acid (cynarin), 1,5-di-*O*-caffeoylquinic acid, 3,5-di-*O*-caffeoylquinic acid, 4,5-di-*O*-caffeoylquinic acid, luteolin-7- rutinoside, luteolin-7-*O*-*β*-D-glucoside (cymaroside), apigenin-7-rutinoside, and apigenin-7-*O*-*β*-D-glucopyranoside [42]. Herein, cynarin and cymaroside are common existing polyphenolic compounds in both flower and bract extracts. In contrast to previous studies, neither caffeic acid nor apigenin were detected in our species. Cynarin is the main derivative of caffeoylquinic acid in leaves and flower extracts of *C. scolymus* L. It was reported that cynarin could reduce the growth of cancerous cells promoting longevity of normal cells [43,44]. Cynaroside has anti-cancer properties through monitoring the processes of cell proliferation, apoptosis, autophagy, invasion, and tumorigenesis [45]. Chlorogenic acid introduces a new strategy in the treatment of cancer through the differentiation of cancer cells rather than killing them [46]. Rutin can act as a chemotherapeutic and chemo-preventive agent that suppresses or prevents either the occurrence of carcinogenesis or the progression of premalignant cells to the invasive stage [47]. Its antitumoral effects can be mediated through the suppression of cell proliferation, the induction of apoptosis or autophagy, and the hindering of angiogenesis and metastasis [48]. Quercetin can prevent and treat different types of cancer by acting as an apoptosis inducer or growth inhibitor [49]. Additionally, catechins showed anticancer activity against various types of cancer through regulation of inflammation, proliferation, cell cycle, oxidative stress, and metastasis processes [50]. So, the isolation of the phenolic fraction of these biologically active compounds from leaf, flower, and stem parts of commercially available artichoke (*C. scolymus* L.) was a must for further assessment of their nanoformulations.

Metal nanoparticles exhibit surface plasmon resonance absorption in the UV-visible range. Because of the small particle size, the surface plasmon band is caused by the continued existence of free electrons in the conduction band [51,52]. This indicates that the Ag^+^ ion has been reduced to colloidal Ag.

TEM was used to show the surface morphology, size distribution, and shape of the produced AgNPs. The image analysis (n = 50 particles) shows a mean particle size of 12.7 nm. This small particle size could enhance the efficacy and cytotoxic effect of the nanoparticles.

The effect of particle size, surface charge, and NP shape on pharmacokinetics, tissue distribution, cellular uptake, and elimination has been extensively proven. Physiological activities such as hepatic absorption and tissue diffusion, tissue extravasation, and renal excretion are also largely dependent on particle size [53]. Furthermore, optimizing the nanocarrier’s particle size, charge, and surface chemistry allows for the avoidance of traditional therapy drawbacks such as excessive dose administration, poor bioavailability, and chemical instability of the delivered medication [54]. The size of AgNPs influences their biological behavior in vivo as well as the nanocarrier’s targeting capabilities [55]. Sriram and his coworkers studied the biologically synthesized AgNPs’ size-dependent cellular cytotoxic effects in primary bovine retinal endothelial cells in vitro. They found that smaller particles of size 22.4 nm, which is comparable with those obtained in this study, exhibited significant toxicity even at the lowest doses compared with the larger size Ag-NPs of size 42.5 nm [56]. 

PDI, on the other hand, is a tool for estimating size distribution and typically ranges from 0 to 1. Low PDI values reflect a limited size distribution and promote long-term nano dispersion stability, but values greater than 0.5 reveal that the size distribution is not uniform [57]. For a lesser fluctuation in AgNPs formulation, lower PDI values are preferred.

The cumulative charges gained by particles are referred to as the zeta potential. It is critical to make precise assessments of the stability of nanoparticle dispersions. Because of electrostatic repulsion between particles, colloidal dispersions with ZP values of 30 mV or above are regarded as highly stable [58]. A high zeta potential will assist the nanocarrier in resisting aggregation by ensuring system stability. When the zeta potential is exceedingly low, the attraction forces outnumber the repulsive forces, causing the dispersion to become unstable. As a result, higher zeta potential nanoparticles are electrically stabilized [59]. 

Some members of the family Compositae are widely used in folk medicine and have been reported to imply a cytotoxic effect against prostate cancer. Extracts of *Vernonia guineensis* Benth., *Melampodium leucanthum*, *Achillea wilhelmsii*, *Achillea teretifolia* Willd, *Gochnatia hypoleuca*, and *Verbesina virginica* were found to exhibit anti-proliferative and cytotoxic activity against the PC-3 and DU145 prostate cancer cell lines through induction of apoptosis or cell cycle arrest [60].

Our biological results indicated that the AgNPs forms of a polyphenolic fraction of flower extract (Nano-TP/Flowers) exhibited potent cytotoxicity against PC-3 and A549 cell lines with IC_50_ values of 0.85 μg/mL and 0.94 μg/mL, respectively, compared with doxorubicin as a standard. Investigating the apoptosis-induction, nano-TP/Flowers exhibited apoptosis in PC-3 with a higher ratio than in A549 cells through flow cytometry and gene expression assays. Our results agreed with their reported anticancer activities [25,61], either through apoptosis-induction or antioxidant activation. A previous study [26] synthesized AgNPs via *Cynara scolymus* leaf extracts and showed their anticancer activity through apoptosis-induction. Mitochondrial pathway activation and Bcl-2 family protein analysis were used to investigate the apoptotic activity. In mitochondria-mediated apoptosis, caspases play critical roles in the initiation and completion of the death process. Enhancement of pro-apoptotic subgroups over anti-apoptotic proteins such as Bcl-2 protein can induce mitochondria to lose mitochondrial potential. The intrinsic apoptotic pathway can be activated by elevating pro-apoptotic proteins over anti-apoptotic ones. This can lead to mitochondria losing their mitochondrial potential (ΔΨm), and releasing cytochrome c., thereby activating cascade reactions of caspase 3 and 9 activations that led to cell death through caspase-dependent apoptosis. So, for the intrinsic pathway, Bcl-2 proteins are essential [62]. These results of AgNP-flower extract are consistent with a recent study showing strong anticancer action of silver nanoparticles functionalized with *Cornus mas* L. extract in an in vivo model through Bcl-2, p53, and metalloproteinase-2 activity, ultimately resulting in apoptosis induction [63]. In the same way that p53 overexpression increases the production of pro-apoptotic proteins such as PUMA, NOXA, and Bax, it also increases apoptotic protein synthesis. For the purpose of mitochondrial membrane instability, these proteins compete with the anti-apoptotic Bcl-2 family members, against which they function as antagonists. On the other hand, the FAS receptor and other surface death receptors are produced thanks to p53, which results in an extrinsic mechanism of cell death. Previous studies on silver nanoparticle-loaded plant extracts found that they activated apoptosis in cancer cell lines, including MCF-7, LNcap, A549, AMJ-13, and THP-1 [64,65,66,67].

Concerning the promising implication of this study, AgNPs proved their successfulness as cytotoxic particles. The green synthesized AgNPs showed the potential of combining the action of both nanoparticles and the naturally occurring agents from the plant extracts. AgNPs are now implemented as biocompatible nanopharmaceuticals, efficient drug delivery vehicles, and biosensors. The use of polyphenols from plant extracts as reducing and capping agents makes eco-friendly delivery systems that are promising in the treatment of lung and prostate cancer which might be effective against other malignancies.

Although AgNPs have been shown to be advantageous for their ease of synthesis, improved efficacy, and potency against cancer cell lines, there are some limitations that should be overcome before they can reach the drug market. Accurate exposure levels of AgNPs for humans and the environment should be optimized. In addition, accurate dosing and accumulation should be further assessed [68].

Finally, these beneficial outcomes recommended further in vivo study of AgNPs of the total phenolic fraction of flower extract of *C. scolymus* L. against an animal model of prostate cancer. Further in-depth estimation of the pharmacokinetics of AgNPs of polyphenols in flower extract will be handled in the future. Moreover, the isolation of other classes of bioactive metabolites combined with molecular docking studies is a topic of interest.

## 4. Materials and Methods

### 4.1. Chemicals 

Cynarin and cynaroside were purchased from Phyproof ^®^, Phyto Lab, Bavaria, Germany, while the remaining herbal reference standards were purchased from Nawah Scientific^®^, Cairo, Egypt. All solvents required for extraction and partitioning processes were of analytical grades. HPLC solvents were purchased from Millipore Sigma (Burlington, MA, USA). Silver nitrate was purchased from Merck (Darmstadt, Germany), ethanol was purchased from Fisher (Waltham, MA, USA), and sodium hydroxide was purchased from Lobechem (Delhi, India). RNeasy™ Mini Kit 50 (QIAGEN, Cat. No. 74104, Germany), i-Script cDNA synthesis kit (BioRad, Cat. No. ♯170-8691, Hercules, CA, USA), and FluoCycle II™ SYBR^®^ Master Mix, Italy, were used. Other chemicals were of analytical grade, and the deionized water was used to wash all the glassware and prepare necessary solutions.

### 4.2. Instruments

A rotary evaporator (Rotavapor R-II, Buchi, Switzerland) was utilized for the solvent evaporation process. The antioxidant assays, as well as quantification assays, were conducted using a spectrophotometer (UV-1601, Shimadzu, Japan). The qualitative identification of phenolic compounds was performed using Waters 2690 Alliance HPLC system and Waters 996 photodiode array detector, Milford, CT, USA. Laminar Flow (model 1386, Thermo Fisher Scientific, Waltham, MA, USA) and BIO-RAD microplate reader (model iMark, Japan) were utilized. Centrifugation of the AgNPs was done using a cooling centrifuge (PRO-Research K241R; Centurion, West Sussex, UK), characterization of the prepared NPs was done using a double-beam spectrophotometer (V630, Jasco, Tokyo, Japan), transmission electron microscope (TEM) (JTEM model 1010, JEOL^®^, Tokyo, Japan) and Malvern Zetasizer (Nano ZS, Malvern Instruments Ltd., Malvern, UK).

### 4.3. Collection of Plant Material and Extraction Process

One kilogram of the plant was purchased from the local Egyptian market in December 2020. The plant was authenticated with the help of the Department of Botany, Faculty of Science, Suez Canal University, Ismailia, Egypt. A voucher specimen was kept at the herbarium of the Pharmacognosy Department, Faculty of Pharmacy, Suez Canal University, under registration code (CS-2020). Three different parts were detached from the plant to be investigated separately: bracts, flowers, and stem. Each part was air dried by placing it on a shallow tray lined with a layer of paper towels in a dry place with good air circulation for 7 to 10 days, and then they were finely ground using an electric grinder. Each part was cold macerated with methanol at room temperature for 1 week. The extraction process was repeated three times to ensure complete extraction. The three extracts were concentrated under reduced pressure using a rotary evaporator to afford crude extracts of different parts: flower (73 g), bract (57 g), and stem (48 g).

### 4.4. In Vitro Antioxidant Activity Assays Crude Extracts of Flower, Bract, and Stem of C. scolymus *L.*

#### 4.4.1. Determination of Total Antioxidant Capacity (TAC) by Phosphomolybdenum Assay

The total antioxidant capacity of crude extracts of different parts was carried out using a phosphomolybdenum assay. This method was carried out as previously mentioned in detail [7,69]. A volume of 0.3 mL of each methanolic crude extract was mixed with 2.7 mL of phosphomolybdenum reagent solution (28 mM sodium phosphate and 4 mM ammonium molybdate in 0.6 M sulphuric acid). Then, the mixture was capped and incubated at 95 °C. After 90 min, the mixture was cooled at ambient temperature, and then the intensity of the green color was recorded spectrophotometrically at 695 nm using methanol as a blank. The experiment was repeated three times. The values were expressed as gallic acid equivalent per gram extract (mg GAE/gm), and ascorbic acid was used as a reference drug.

#### 4.4.2. Ferric Reducing Antioxidant Power (FRAP) Assay

The reducing potential of crude extracts of different parts was assessed by mixing 2 mL of each crude extract with 2 mL of sodium phosphate buffer solution (0.2 M; pH = 6.6) and 2 mL of potassium ferricyanide (10 mg/L). The reaction mixture was incubated for 20 min at 50 °C and then acidified using 2 mL of trichloroacetic acid (100 mg/L). After centrifugation, 2 mL of the supernatant was picked up, and diluted with 2 mL of distilled water and 0.4 mL of ferric chloride (0.1%). After 10 min, the intensity of color was measured spectrophotometrically using at 700 nm using methanol as a blank [7,69]. The percent FRAP activity was calculated according to the following formula: %FRAP activity = [(A_n_ − A_s_) × 100]/A_n_, where A_n_ is the final absorbance value of negative control, and A_s_ is the final absorbance value of samples. The value of IC_50_ (the concentration that is required to reduce the initial concentration of Fe^3+^ ions by 50%) was determined by a linear concentration/percentage inhibition curve with a correlation coefficient (R^2^) of 0.993. The experiment was also done in triplicate. Ascorbic acid was used as a reference drug and applied at the same concentrations of the sample, 2.5, 5, 10, 20, 40, 80, 160, 320, 640, and 1280 µg/mL.

#### 4.4.3. DPPH Radical Scavenging Assay

The free radical scavenging activity of crude extracts of different parts was evaluated via mixing equal volumes of each sample with 1,1-diphenyl-2-picrylhydrazyl (DPPH) free radical (200 μM). The reaction mixture was incubated for 30 min in dark conditions at 30 °C. The intensity of the yellow color was measured spectrophotometrically at 516 nm using methanol as a blank. The experiment was also done in triplicate. The scavenging percentage was calculated based on the following equation: [(A_control_ − A_sample_)/A_control_ × 100], where A _control_ is referred to the absorbance of the DPPH solution only, and A_sample_ is referred to the absorbance of both DPPH solution and sample at different concentrations (5–1000 µg/mL) [7,69]. The value of IC_50_ (the concentration that is required to inhibit the initial concentration of DPPH by 50%) was determined by a linear concentration/percentage inhibition curve (R^2^ = 0.998). Ascorbic acid was used as the reference standard and applied at the same concentrations of the sample, 2.5, 5, 10, 20, 40, 80, 160, 320, 640, and 1280 µg/mL. 

### 4.5. Spectrophotometric Quantification of Total Phenolics Content and Total Flavonoids Content in Crude Extracts of Flower, Bract, and Stem of C. scolymus *L.*

#### 4.5.1. Estimation of Total Phenolic Content Using Folin–Ciocalteu Method

The total phenolic content in different parts of *C. scolymus* L. was determined spectrophotometrically using Folin–Ciocalteu (Folin-C) method. As mentioned before [5], each sample (500 µL) was mixed with Folin-C reagent (2.5 mL, 10%) and sodium carbonate (2 mL, 7.5%). The reaction was stored for 2 h in a dark condition at room temperature. The intensity of the blue color was measured spectrophotometrically at 760 nm using distilled water as a blank. The experiment was replicated three times. The total phenolic content values are expressed as gallic acid equivalent per gram extract (mg GAE/gm).

#### 4.5.2. Estimation of Total Flavonoids Content Using Aluminum Complexation Method

Concerning estimation of total flavonoid content in crude extracts of flower, bract, and stem, an aluminum complexation reaction was done [5]. One mL of sample was mixed with 0.3 mL of sodium nitrite solution (5%), 4 mL of distilled water, and 0.3 mL of AlCl_3_.6H_2_O solution (10%). The mixture was incubated for 5 min at 25 °C. At last, 2 mL of 1 M NaOH solution was added, and the total volume was completed to 10 mL with distilled water. The intensity of yellowish-orange color was measured at 510 nm against distilled water as a blank. In addition, the experiment was repeated three times. The values of total flavonoid content are expressed as quercetin equivalent per gram extract (mg QE/gm).

### 4.6. HPLC-DAD Identification of Polyphenols in Crude Extracts of Flower, Bract, and Stem of C. scolymus *L.*

For comparative assessment of polyphenols in crude extracts of different parts, gallic acid, chlorogenic acid, ellagic acid, catechins, cynarin, cymaroside, rutin, hesperidin, quercetin, kaempferol, and apigenin were purchased. Chemical profiling was performed using high-performance liquid chromatography combined with a diode array detector (HPLC-DAD). First, a standard mixture of all reference polyphenols was prepared, and an injection volume of10 µL was applied into the C18 column Inertsil ODS (4.6 × 250 mm, 5 µm). The mobile phase consisted of 0.1% phosphoric acid in water: acetonitrile with a constant flow rate of 1 mL/minute and a pH of 3.5 [5]. The absorbance was measured at 280 nm. Then, 100 mg of the methanolic crude extracts of flower, bark, and stem was accurately weighed, dissolved in 100 mL methanol, sonicated for 15 min, filtered through a 0.22 µm Nylon syringe filter, and then an amount of 10 µL was injected.

### 4.7. Preparation of Phenolic Portions of Flower, Bract, and Stem of C. scolymus *L.*

The phenolic portion was extracted from different parts by suspending 2 gm of different extracts of flower, bract, or stem with 100 mL of an aqueous solution of 5% sodium carbonate for one hour with the aid of sonication to ensure the acid-base reaction process. Then, the reaction mixture was filtered and washed with distilled water for complete extraction [69,70]. The aqueous solution was partitioned with *n*- butanol to extract a non-phenolic portion. The remaining aq. solution was neutralized by hydrochloric acid using litmus paper as an indicator. Finally, the phenolic portion was extracted with ethyl acetate three times by liquid-liquid extraction and concentrated under a vacuum. The total phenolic fractions of flower (300 mg), bract (180 mg), and stem (170 mg) were obtained.

### 4.8. Formulation of Silver Nanoparticles (AgNPs) of Different Phenolic Portions

#### 4.8.1. Preparation of Silver Nanoparticles

The synthesis of AgNPs using a biogenic pathway in the presence of the phenolic extracts of flower, bract, and stem was prepared using a modified method that was previously reported [71,72,73]. Initially, 10 mg of the extract was dissolved in 1 mL ethanol, then added to 10 mL of 10 mM AgNO_3_. A few drops of 1 M NaOH were added, and the mixture was agitated for 1 h at 400 rpm at 60 °C in the dark. All prepared nanoparticles were purified by centrifugation at 15,000 rpm for 1 h at 4 °C. The AgNPs were re-dispersed in double-distilled water and sonicated for 30 s in a sonicating water bath, then centrifuged under the same previous conditions. The washing procedures using double-distilled water were repeated three times.

#### 4.8.2. Characterization of Silver Nanoparticles

- UV-VIS Spectroscopy.

The reduction of Ag^+^ ion was verified by using a double-beam spectrophotometer to analyze the UV-vis spectrum. The spectra were captured over a wavelength range of 300–600 nm.

- Transmission electron microscopy (TEM).

The size and surface appearance of the produced AgNPs were examined using TEM. The sample preparations were then diluted with double distilled water 50 times. The diluted samples were then stained negatively with phosphotungstic acid before being dried on carbon-coated copper grids. The thin film was produced and examined using a transmission electron microscope with an accelerating voltage of roughly 80 kV [74]: particle size and zeta potential determination.

Photon correlation spectroscopy (PCS) was used to correctly quantify average particle size (Z-average), zeta potential (ZP), and polydispersity index (PDI) using Malvern Zetasizer. Before analysis, each sample was diluted 20 times with distilled water. All measurements were carried out in triplicates at room temperature (25 °C). Finally, the mean and standard deviation were determined with precision [75]. 

### 4.9. Comparative Assessment of In Vitro Cytotoxic Activity

#### 4.9.1. MTT Assay

Lung (A549) and prostate (PC-3) cancer cell lines were obtained from the National Cancer Institute in Cairo, Egypt, cultured on Dulbecco’s Modified Eagle Medium and Roswell Park Memorial Institute Medium (RPMI-1640/DMEM) supplemented with L-glutamine (Lonza Verviers SPRL, Belgium, cat#12-604F). The cells were cultured in 10% fetal bovine serum (FBS, Sigma-Aldrich, City of Saint Louis, MO, USA) and 1% penicillin-streptomycin (Lonza, Belgium). All cells were incubated at 37 °C in a 5% carbon dioxide atmosphere (NuAire). In a 96-well plate, cells were plated in triplicate at a density of 5 × 10^4^ cells. Then, they were treated with different total phenolic portions (TP/Flower, TP/Bract, TP/Stem) and their nano forms (Nano-TP/Flower, Nano-TP/Bract, Nano-TP/Stem) at concentrations of (0.1, 1, 10, and 100 μg/mL) on the second day. Cell viability was assessed using an MTT solution (Promega, Madison, WI, USA) [76]. Three hours were spent incubating the plate. The absorbance was then measured with an ELISA microplate reader (BIO-RAD, model iMark, Japan). The viability was calculated compared with control, and the IC_50_ values were computed using the GraphPad prism 7 (Dotmatics, San Diego, CA, USA) as previously reported [76,77].

#### 4.9.2. Investigation of Apoptosis

- Annexin V/PI staining and cell cycle analysis

Both PC-3 and A549 cells were incubated into 6-well culture plates (3–5 × 10^5^ cells/well) overnight, and they were then treated with a total phenolic portion of flower part (Nano-TP/Flower) at a dose equal to its IC_50_ of each cell line (IC_50_ = 0.85µM, 48 h) and (IC_50_ = 0.94 µM, 48 h), respectively. Then, media supernatants and cells were collected. The cells were suspended in 100 µL of Annexin binding buffer solution “25 mM CaCl_2_, 1.4 M NaCl, and 0.1 M Hepes/NaOH, pH 7.4” and incubation with “Annexin V-FITC solution (Sigma Aldrich, City of Saint Louis, MO, USA) (1:100) and propidium iodide (PI) (Sigma Aldrich, USA) at a concentration equals 10 µg/mL in the dark for 30 min.” The stained cells were then harvested using the Cytoflex FACS machine, and cytExpert software (Beckman Coulter, Brea, CA, USA) was used to analyze the data [78,79].

- Gene expression analysis (RT-PCR) for the selected genes

To further investigate the apoptotic pathway, we followed the gene expression of P53, Bax, Caspapses-3, 8, 9 as pro-apoptotic genes and Bcl-2 as the anti-apoptotic gene using the RT-PCR system (MiniOpticon™, Cycler, Singapore); their sequences in forward and reverse direction are expressed in Table 5. 

PC-3 cells were treated with a sample of Nano-TP/Flowers (IC_50_ = 0.85 µM, 48 h). Then, an RT-PCR reaction was performed following routine work, and the results were given in cycle thresholds (Ct) and ΔΔ Ct for calculating the relative quantities of each gene to the housekeeping gene (β-actin) as previously described [80,81,82]. 

## 5. Conclusions

In conclusion, the flower part of *C. scolymus* L. is a rich source of polyphenolic compounds and antioxidant compounds compared with other edible leaf and stem parts. The polyphenolic compounds of these edible parts were compared with each other using HPLC-DAD. Silver nanoparticles of polyphenolic fractions of flower, leaf, and stem parts were developed and characterized. Finally, AgNPs of the phenolic fraction of the flower part exhibited potent cytotoxic activity against the prostate PC-3 cancer cell line. Herein, the AgNPs forms of the total phenolic fraction of *C. scolymus* L. are introduced as adjuvant therapy in the treatment of prostate cancer.

## Figures and Tables

**Table 1:** Total antioxidant capacity, ferric reducing antioxidants power, and DPPH radical scavenging assays of flower, bract, and stem of *C. scolymus* L.

Sample	TAC Assay (mg GAE/g)	IC_50_ of FRAP Assay (µg/mL)	IC_50_ of DPPH Scavenging Activity (µg/mL)
Crude extract of Flower part	34.07 ^a^ ± 3.15	77.12 ^c^ ± 4.23	45.91 ^c^ ± 2.97
Crude extract of Bract part	32.13 ^a^ ± 2.49	91.11 ^b^ ± 6.17	64.39 ^b^ ± 3.45
Crude extract of Stem part	28.16 ^a^ ± 2.34	604.13 ^a^ ± 17.85	514.02 ^a^ ± 9.86
Ascorbic acid	2.49 ^b^ ± 3.91	17.11 ^d^ ± 0.90	10.65 ^d^ ± 0.83
ANOVA (*p*-value)	<0.001 ***	<0.001 ***	ANOVA (*p*-value)

TAC: total antioxidant capacity; FRAP: ferric reducing antioxidant power; DPPH: 1,1-diphenyl-2-picrylhydrazyl; GAE: gallic acid equivalent. *** Significant at *p* < 0.001. Means followed by different letters (a, b, c, d) are significantly different according to One-way ANOVA in GraphPad Prism software.

**Table 2:** Total phenolics and total flavonoids in flower, bract, and stem of *C. scolymus* L.

Sample	Total Phenolic (mg GAE/gm)	Total Flavonoids (mg QE/gm)
Crude extract of Flower part	74.29 ^a^ ± 3.85	46.03 ^a^ ± 1.99
Crude extract of Bract part	60.94 ^b^ ± 3.28	21.89 ^b^ ± 1.07
Crude extract of Stem part	26.59 ^c^ ± 1.37	8.26 ^c^ ± 0.92
ANOVA (*p*-value)	<0.001 ***	<0.001 ***

GAE: gallic acid equivalent; QE: quercetin equivalent. *** Significant at *p* < 0.001. Means followed by different letters (a, b, c) are significantly different according to One-way ANOVA in GraphPad Prism software.

**Table 3:** Particle size (PS), polydispersity index (PDI), and zeta potential (ZP) of the synthesized AgNPs.

Formula	PS (nm)	PDI	ZP (mV)
AgNPs of total phenolics of flower (Nano-TP/Flower)	21.31 ± 0.431	0.109 ± 0.014	−34.0 ± 4.45
AgNPs of total phenolics of bract (Nano-TP/Bract)	22.05 ± 0.912	0.101 ± 0.017	−35.5 ± 2.69
AgNPs of total phenolics of stem (Nano-TP/Stem)	26.42 ± 1.082	0.112 ± 0.020	−31.9 ± 2.22

**Table 4:** IC_50_ values of different fractions of total phenolics and their AgNPs forms of different parts.

Samples	Working Concentration	IC_50_ * [μg/mL]	
		PC-3	A549
TP/Flower	0.1, 1, 10, 50, 100 μg/mL	16.35 ± 0.76	17.38 ± 0.75
TP/Bract		19.65 ± 0.97	21.04 ± 0.96
TP/Stem		43.2 ± 1.51	56.3 ± 2.12
Nano-TP/Bract		1.01 ± 0.1	1.34 ± 0.23
Nano-TP/Flowers		0.85 ± 0.01	0.94 ± 0.02
Nano-TP/Stem		14.3 ± 0.43	13.6 ± 0.34
Doxorubicin		5.13 ± 0.64	6.19 ± 0.58

* IC_50_ were calculated by non-linear regression curve fir using GraphPad prism; TP: total phenolic fraction.

**Table 5:** Sequences of forward and reverse primers.

Gene	Forward	Reverse
P53	5′-CCCCTCCTGGCCCCTGTCATCTTC-3′	5′-GCAGCGCCTCACAACCTCCGTCAT-3′
Bax	5′-GTTTCATCCAGGATCGAGCAG-3′	5′-CATCTTCTTCCAGATGGTGA-3′
CASP-3	5′-TGGCCCTGAAATACGAAGTC-3′	5′-GGCAGTAGTCGACTCTGAAG-3′
CASP-8	5′-AATGTTGGAGGAAAGCAAT-3′	5′-CATAGTCGTTGATTATCTTCAGC-3′
CASP-9	5′-CGAACTAACAGGCAAGCAGC-3′	5′-ACCTCACCAAATCCTCCAGAAC-3′
Bcl-2	5′-CCTGTGGATGACTGAGTACC-3′	5′-GAGACAGCCAGGAGAAATCA-3′
β-actin	5′-GTGACATCCACACCCAGAGG-3′	5′-ACAGGATGTCAAAACTGCCC-3′

## Data Availability

Data are available within the article.

## Samples Availability

Cynarin and cymaroside were purchased from Phyproof^®^, Phyto Lab, Bavaria, Germany, while the remaining herbal reference standards were purchased from Nawah Scientific^®^, Cairo, Egypt.
